# Lack of retinoid acid receptor-related orphan receptor alpha accelerates and melatonin supplementation prevents testicular aging

**DOI:** 10.18632/aging.103654

**Published:** 2020-07-09

**Authors:** Ramy K. A. Sayed, Doaa M. Mokhtar, Marisol Fernández-Ortiz, José Fernández-Martínez, Paula Aranda-Martínez, Germaine Escames, Darío Acuña-Castroviejo

**Affiliations:** 1 Instituto de Biotecnología, Centro de Investigación Biomédica, Parque Tecnológico de Ciencias de la Salud, Universidad de Granada, Granada 18016, Spain; 2 Department of Anatomy and Embryology, Faculty of Veterinary Medicine, Sohag University, Sohag 82524, Egypt; 3 Department of Anatomy and Histology, Faculty of Veterinary Medicine, Assiut University, Assiut 71526, Egypt; 4 Departamento de Fisiología, Facultad de Medicina, Universidad de Granada, Granada 18016, Spain; 5 CIBER de Fragilidad y Envejecimiento, Ibs. Granada, Unidad de Gestión Clínica de Laboratorios Clínicos, Hospital Universitario San Cecilio, Granada 18016, Spain

**Keywords:** aging, Leydig cells, seminiferous tubules, RORα, Sertoli cell

## Abstract

The role of retinoid acid receptor-related orphan receptor alpha (RORα) on male reproductive functions during aging is unclear. Here, we analyze the morphological changes in the testis of both young and aged RORα-deficient mice, with and without melatonin supplementation. Young mutants showed vacuolation, degeneration and pyknosis of spermatogenic epithelium and Sertoli cells. Aged mutants showed atrophy of the seminiferous tubules and absence of mitotic spermatogenic cells. Absence of sperms in many tubules, loss of acrosomal cap, vacuolation and hypertrophy of Sertoli cells were detected in aged mice, with a significant reduction in the number of seminiferous tubules and a significant increase in the number of Leydig cells and telocytes. Repair in seminiferous tubules and interstitial tissues with enhancement of spermatogenesis was observed in melatonin-treated aged mice. Young mutants overexpressed VEGF that was weaker in aged animals and observed only in the spermatocytes, while melatonin increased VEGF expression in spermatocytes and spermatids. Caspase 3 increased in both young and aged mutant mice in all seminiferous tubules and interstitium; caspase 3 immunostaining in seminiferous tubules, however, showed a normal pattern of apoptosis with melatonin supplementation. The present study reports that age-dependent testicular changes in RORα mutant mice were recovered by melatonin treatment.

## INTRODUCTION

Aging is a complex phenomenon that results from accumulations of various adverse alterations including genetic and developmental defects, environmental, epigenetic and stochastic events, disease and innate aging process [1]. It is associated with an irreversible and progressive decline of the body functions due to biochemical and morphological changes, and consequently increases death risk. However, many theories have been progressed to explain aging process; none is fully accepted [2].

Aging induces decline of male reproductive functions as a result of morphological as well as molecular changes in the genital organs and so promotes alterations in hormones levels. This decline extends to sperm quality and production, and also to the histological structure of reproductive organs [3]. Testis is the main genital organ responsible for spermatogenesis and production of testosterone male hormone, and both functions have strong relation and are affected by aging process [4, 5]. Among morphological changes occurring in testis with aging are the reductions of germ cells volume and number as well as decline of sperm quality and fertility [6, 7].

Retinoid acid receptor-related orphan receptor alpha (RORα) is one of nuclear orphan receptors family, which plays a role in many processes including cellular function, lipid homeostasis and tissue development and differentiation [8–10]. Recently, many experimental studies revealed the crucial roles of RORα on maturation, development and neuroprotection of different cerebral regions, where RORα activity is an important indicator for detection and avoiding emergence of special brain infections [11]. Moreover, RORα1 was recently detected to have an essential role in breast cancer cells and its overexpression induces proliferation of these cells [12].

In male genital system, previous studies reported RORα expression before puberty in the epididymis and after sexual maturation in the peritubular cells of testis [13]. Our previous study proposed the role RORα in regulation of testicular structure and revealed many testicular changes in 3 month-old RORα^-/-^ mice comparing with wild-type mice. These changes include hypospermatogenesis, reduction of Leydig cells number, abnormal Sertoli cells, vacuolation of seminiferous tubules epithelium, signs of delay spermiation with abnormal sperm morphology and ill-developed sperms [14].

Melatonin, or N-acetyl-5-methoxytryptamine (aMT), is a hormone that is produced in the pineal gland [15] and also in various tissues and organs including testis [16–18]. It has powerful anti-inflammatory and anti-oxidative effects [19–21], as well as its role in reduction of free radicals formation, induction of ATP production [22, 23], maintaining mitochondrial function integrity, and preventing mitochondrial damage during aging [24–27]. Recent studies reported beneficial effects of melatonin in prevention and treatment of age-related macular degeneration [28], induction of cellular autophagic repair, regulation of inflammatory and immune responses, alleviation of endoplasmic reticulum stress and protection of cardiomyocytes from acute infarction [29]. Furthermore, melatonin was recently detected to prohibit invasion and proliferation of melanoma cells controlling their growth [30]. In male reproductive organs, melatonin modulates the endocrine activities in Leydig and Sertoli cells, affects cellular growth and proliferation, as well as energy metabolism, and consequently plays a vital role in regulation of spermatogenesis [31].

The crucial roles of RORα on male reproductive functions, specifically on morphological and structural elements of the testis during aging are not fully understood. Thus, this study highlights the histological, histochemical and ultrastructural changes in the testicular cells of both young and aged RORα-deficient mice, as well as investigating the protective effect of melatonin supplementation on these changes.

## RESULTS

### Anthropometric analysis of body weight and testicular weight

Body and testicular weights of the control mice was 24.9 ± 2.17 g and 97.78 ± 6.27 mg respectively. Young mutant mice reported significant declines in body weight (18.76 ± 1.23 g, *p* < .05) and testicular weight (62.73 ± 6.84 mg, *p* < .05), associated with significant decrease in the ratio of the testicular weight to the body weight from 0.39 ± 0.01 % in the control mice to 0.33 ± 0.02 % in the young mutant ones.

**Figure 1:**
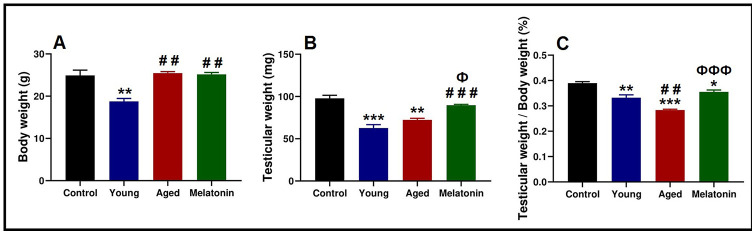
**Anthropometric analysis of the body weight and testicular weight in the young and aged mice.** (**A**) Analysis of body weight in control, young mutant, aged mutant and aged mutant with melatonin. (**B**) Analysis of the testicular weight. (**C**) Ratio of the testicular weight to the body weight. * *p*<.05, ** *p*<.01 and *** *p*<.001 *vs.* Control; ## *p*<.01 and ### p<.001 *vs.* Young; Ф *p*<.05 and ФФФ *p*<.001 *vs.* Aged.

With aging, mutant mice showed a significant increase in the body weight (25.47 ± 0.60 g, *p* < .05), associated with non-significant increase in the testicular weight (72.33 ± 3.15 mg); however, the ratio of the testicular weight to the body weight was significantly decreased (0.28 ± 0.01 %, *p* < .05). Melatonin supplementation reported non-significant changes in the body weight (25.17 ± 0.78 g), with a significant induction of testicular weight increase (89.74 ± 1.58 mg, *p* < .05). Moreover, melatonin supplementation induced the ratio of testicular weight to body weight (0.36 ± 0.01 %, *p* < .05, Figure 1A–1C).

### Histological analysis

**Figure 2:**
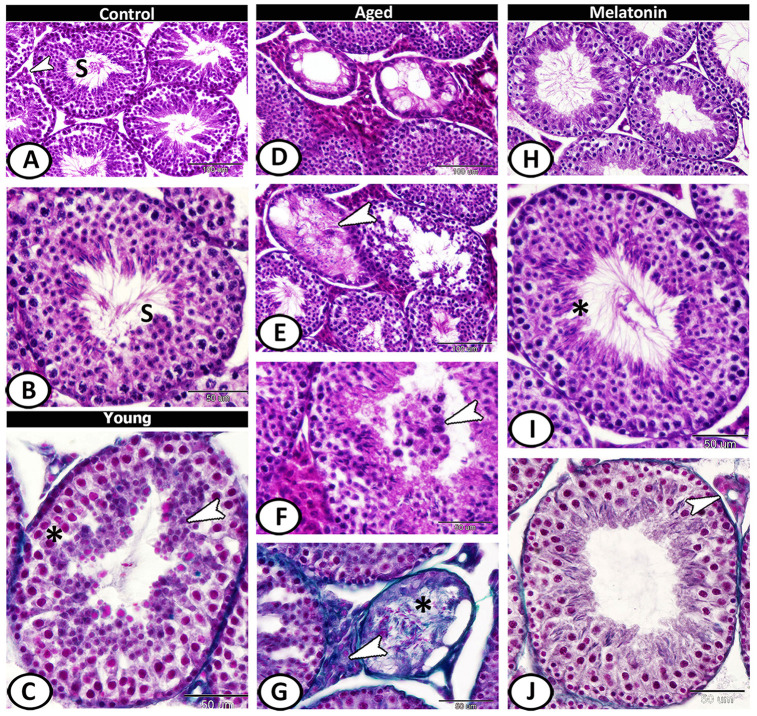
**Histological pattern of spermatogenesis in young and aged mice stained with HE except for C, G, J are stained with Crossmon's trichrome.** (**A**, **B**) Seminiferous tubules of the control group characterized by normal spermatogenesis (S). Note, a narrow interstitium contained interstitial cells (arrowhead). (**C**) Seminiferous tubules in young mutant mice characterized by pyknosis (asterisk) in the spermatogenic epithelium with spermatids aggregation (arrowhead). (**D**) In aged mice, a mosaic pattern of seminiferous tubules ranged from tubules with complete, reduced spermatogenesis to degenerated tubules. (**E**) Clusters of vacuoles and absence of mitotic cells among the epithelium of seminiferous tubules with apparent degeneration, and absence of sperms in many tubules of aged mice (arrowhead). (**F**) The lumen of seminiferous tubules contained spermatids and spermatocytes (arrowhead). (**G**) The volume of interstitial tissue increased with an abundance of collagen fibers (arrowhead). Note sclerosed tubule (asterisk) that showed loss of spermatids. (**H**–**J**) In melatonin group, enhanced spermatogenesis, presence of many sperms (asterisk) and normal interstitial tissues (arrowhead) demonstrated.

The seminiferous tubules of the control group were characterized by a normal arrangement of their cellular components that separated by a narrow interstitium contained interstitial cells (Figure 2A, 2B). In young mutant mice, vacuolation, degeneration, and pyknosis in the spermatogenic epithelium were evident with spermatids aggregation (Figure 2C). The aged mice showed a mosaic pattern of seminiferous tubules ranged from tubules with complete, reduced spermatogenesis to degenerated tubules. Most of the tubules showed atrophy, clusters of vacuoles among the spermatogenic epithelium (Figure 2D). The spermatogenic cells showed an absence of mitotic cells, with apparent degeneration, and absence of sperms in many tubules (Figure 2E). Dark eosin-stained cells with pyknotic nucleus may correspond to apoptotic cells were observed and the lumen of some seminiferous tubules contained spermatids and spermatocytes (Figure 2F). The volume percentage of interstitial tissue increased with an abundance of collagen fibers (sclerosed tubules). The atrophied tubules were observed in patches and showed loss of spermatids and formed of spermatocytes or only spermatogonia. Most of the seminiferous tubules were covered by thick basement membrane (Figure 2G). In the melatonin group, an evident and sufficient repair in seminiferous tubules and interstitial tissues occurred with enhanced spermatogenesis (Figure 2H–2J).

Morphometrically, the diameter of the seminiferous tubules and height of their epithelium revealed a significant decline from 178.62 ± 4.65 μm and 58.65 ± 3.42 μm in the control mice, respectively, to 152.67 ± 4.97 μm and 45.21 ± 2.81 μm in the young mutant mice, respectively, with non-significant decrease in the number of seminiferous tubules (7.25 ± 0.95 in the control mice and 5.25 ± 1.5 in the mutant ones). With aging, the number and diameter of seminiferous tubules as well as height of their epithelium were significantly decreased to about 4.5 ± 1.29, 139.19 ± 11.94 μm and 36.11 ± 5.47 μm respectively; however, these reductions were significantly counteracted with melatonin supplementation to 8 ± 0.82, 192.87 ± 8.86 μm and 65.77 ± 4.73 μm respectively (Supplementary Figure 1A–1C). The volume percentage of interstitial tissue in the control and young mutant mice were 46.23 ± 3.67 % and 40.53 ± 4.43 % respectively. With aging, there was a significant increase in this volume to 53.75 ± 2.40 %, but this increase reduced significantly with melatonin supplementation to 33.60 ± 5.26 % (Supplementary Figure 1D).

**Figure 3:**
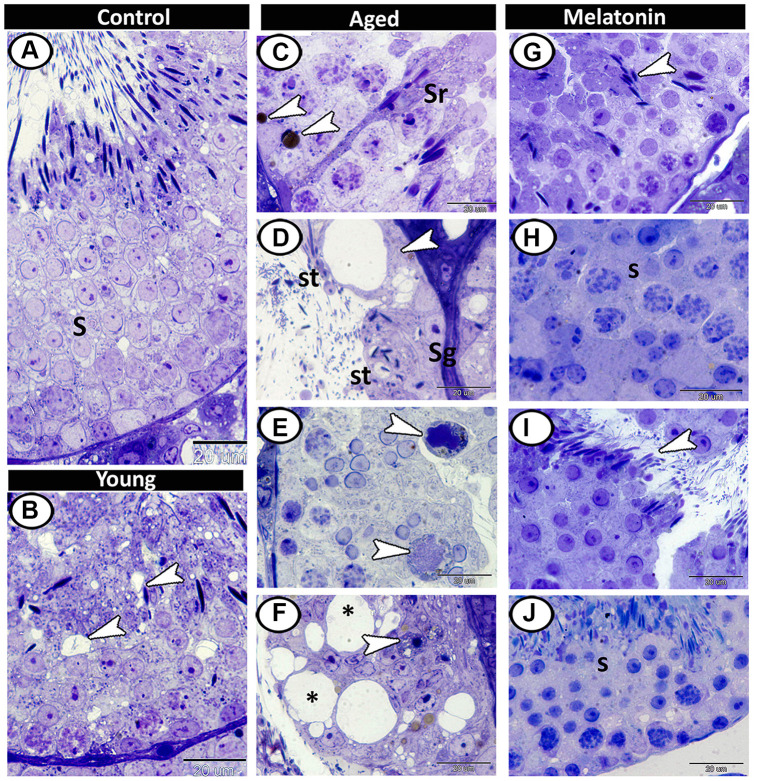
**Semithin section of seminiferous tubules in the young and aged mice.** (**A**) Different stages of spermatogenesis (S) in the control group. (**B**) In the young mutant mice, the presence of small vacuoles between the spermatogenic cells was observed (arrowheads). (**C**) The aged testis characterized by the presence of lipofuscin pigments (arrowheads) and Sertoli cells hypertrophy (Sr). (**D**) Vacuolation in Sertoli cells (arrowhead), many of seminiferous tubules only contained spermatogonia (Sg) and spermatids (St). (**E**) Multinucleated giant cells (arrowheads) were frequently demonstrated in the seminiferous tubules. (**F**) Some degenerated spermatogonia with pyknotic nuclei (arrowhead) were observed with vacuolated spermatogenic cells (asterisks). (**G**–**J**) In melatonin treated group, the seminiferous tubules showed normal spermatogenesis (S), and many mature sperms (arrowheads).

Semithin section showed different stages of spermatogenesis in the control group (Figure 3A), while the presence of small vacuoles between the spermatogenic cells was the characteristic feature in the young mutant mice (Figure 3B). At the aging group, the presence of lipofuscin pigments and Sertoli cells hypertrophy were demonstrated (Figure 3C), as well as vacuolation with depletion in spermatogenic cells were evident so many of seminiferous tubules only contained spermatogonia and spermatids (Figure 3D). Multinucleated giant cells were frequently demonstrated in the lumen of seminiferous tubules and among the spermatogenic epithelium (Figure 3E). Loss of sperms in some tubules and some spermatogonia degenerated with pyknotic nuclei were also recorded in aged testis (Figure 3F). In melatonin treated group, the seminiferous tubules showed rearrangement of spermatogenic cells, normal spermatogenesis, and the presence of many mature sperms (Figure 3G–3J).

**Figure 4:**
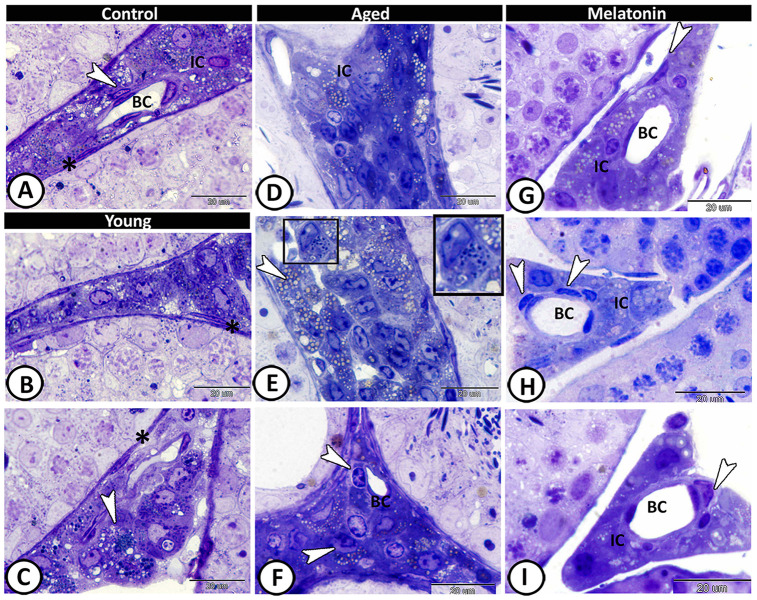
**Semithin section of the interstitium in the young and aged mice.** (**A**) In the control group, the interstitium contained Leydig cells (IC), myoid cells (asterisk), telocytes (arrowhead) and blood capillaries (BC). (**B**, **C**) The young mutant mice showed many lipid droplets in the Leydig cells (arrowhead), and normal myoid cells (asterisks). (**D**, **E**) Significant increase in the number of Leydig cells and their lipid droplets (arrowheads, IC) was detected in the aged mice. Note the presence of mast cells (boxed areas). (**F**) Dendritic reticular cells (arrowheads) were recorded around the blood capillaries (BC), among the affected interstitial cells (IC). (**G**–**I**) In melatonin group, the Leydig cells (IC) return to their normal number with widening in the lumen of blood capillaries (BC) and increase in the number of the telocytes (arrowheads).

In the control group, the interstitium contained Leydig cells, myoid cells, telocytes and blood capillaries (Figure 4A). The young mutant mice showed many lipid droplets in the Leydig cells (Figure 4B, 4C). A significant increase in the number of Leydig cells (4.66 ± 0.58 in the control mice, 2.67 ± 0.5 in the mutant mice and 7.67 ± 1.15 in the aged one, Supplementary Figure 1E), and their lipid droplets were evident in the aged mice (Figure 4D, 4E). Furthermore, mast cells and dendritic reticular cells were recorded among the affected interstitial cells (Figure 4E, 4F). In melatonin group, the Leydig cells return to their normal number (3.33 ± 1.53, Supplementary Figure 1E), and the interstitium showed widening in the lumen of blood vessels, associated with an increase in the number of the telocytes (Figure 4G–4I).

### Histochemical analysis and enzyme histochemistry

**Figure 5:**
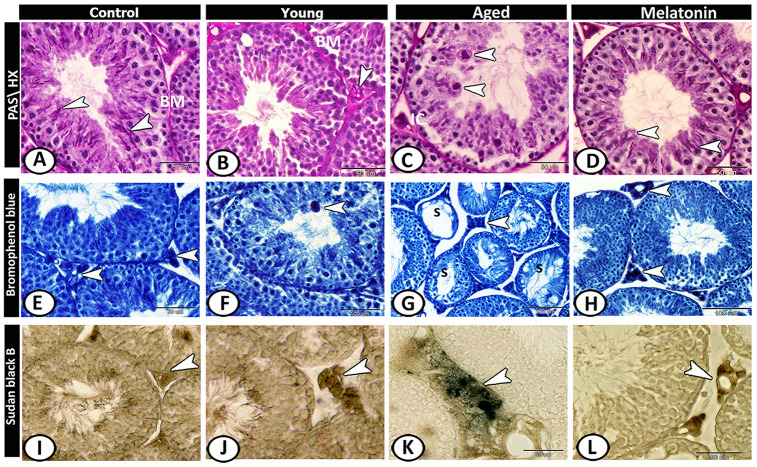
**Histochemical analysis of testis of the young and aged mice.** (**A**) In the control group, the basement membrane (BM) of seminiferous tubules, Sertoli cells and sperms (arrowheads) showed staining affinity to PAS. (**B**) In the young mutant mice, the basement membrane (BM) appeared thick and PAS-positive and the interstitium is PAS-positive (arrowhead). (**C**) In aged mice, Leydig cells (IC) and giant cells (arrowheads) in the seminiferous tubules showed strong PAS-positive reactions. (**D**) In melatonin group, Sertoli cells and their associated spermatids (arrowheads) showed PAS-positive reaction. (**E**) In the control group, the Leydig cells (arrowhead) showed affinity to bromophenol blue (BB). (**F**) In young mutant mice, some giant cells (arrowhead) reacted positively to BB. (**G**) In aged mice, the degenerated seminiferous tubules (S) showed no or weak affinity to BB, while Leydig cells (arrowhead) showed strong BB- positive reactions. (**H**) In the melatonin group, the reaction was concentrated in Leydig cells (arrowheads). (**I**) Sudan black B stain showed a weak reaction in Leydig cells (arrowhead) of the control group. (**J**) A moderate reaction in Leydig cells (arrowhead) of young mice. (**K**) An evident increase in the number of positive- lipid droplets in interstitial cells (arrowhead) was observed in aged mice. (**L**) The reaction (arrowhead) was moderate in the melatonin group.

In the control group, the basement membrane of seminiferous tubules, Sertoli cells, and sperms showed staining affinity to PAS (Figure 5A). In the young mutant mice, the basement membrane appeared thick and PAS-positive and the interstitium showed high affinity to PAS (Figure 5B). In aged mice, Leydig cells showed strong PAS-positive reactions with the presence of many PAS-positive giant cells as well as the number of PAS-positive sperms was much reduced (Figure 5C). In the melatonin group, the reaction was well-defined in the Sertoli cells and their associated spermatids (Figure 5D).

Leydig cells of control animals showed affinity to bromophenol blue (BB) (Figure 5E). In young mutant mice, some giant cells reacted positively to BB (Figure 5F). In aged mice, the degenerated seminiferous tubules showed no or weak affinity to BB, while Leydig cells showed strong BB- positive reactions (Figure 5G). This reaction in melatonin group was concentrated in Leydig cells (Figure 5H).

Sudan black B stain showed weak reaction in Leydig cells of the control group (Figure 5I), moderate in young mice (Figure 5J) and an evident increase in the number of positive lipid droplets in interstitial cells was observed in aged mice (Figure 5K), while the reaction was moderate in the melatonin group (Figure 5L).

**Figure 6:**
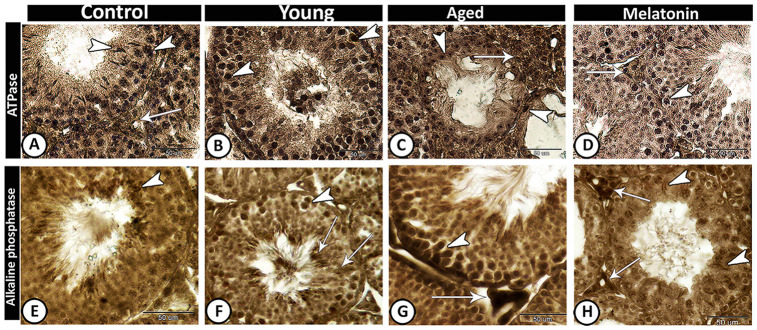
**Enzyme histochemistry in the young and aged mice.** (**A**, **D**) In both control and melatonin groups, ATPase activities increased in Leydig cells (arrow) and active germ cells (arrowheads). (**B**) ATPase activities were concentrated in spermatogonia and spermatocytes (arrowheads) in young mutant mice. (**C**) In the aged mice, the staining reaction increased in interstitial cells (arrow) and some spermatogonia (arrowheads). (**E**) In control, alkaline phosphatase staining was detected in the elongated spermatids (arrowhead). (**F**) In young mutant mice, the reaction was intense in the germ cells (arrowhead) and Sertoli cells and their associated spermatids (arrows). (**G**) In aged mutant mice, the activity increased in spermatogonia and spermatocytes (arrowhead) and the Leydig cells showed high acid phosphatase activity (arrow). (**H**) In the melatonin group, alkaline phosphatase activity was detected in the active Sertoli cells (arrowheads) and Leydig cells (arrows).

In control and melatonin groups, ATPase activities increased in Leydig cells and active germ cells (Figure 6A, 6D), while its activities were concentrated in spermatogonia and spermatocytes of young mutant mice (Figure 6B). In the aged mice, the staining reaction increased in interstitial cells and decreased in seminiferous tubules except for some spermatogonia (Figure 6C). In the melatonin group, ATPase activities were evident in spermatogonia and spermatocytes as well as interstitial cells.

The alkaline phosphatase staining reaction was detected in control animals in the elongated spermatids (Figure 6E), while in young mutant mice the reaction was intense in the germ cells and Sertoli cells and their associated spermatids (Figure 6F). In aged mutant mice, the alkaline phosphatase activity increased in spermatogonia and spermatocytes as well as the Leydig cells showed high activity (Figure 6G). In the melatonin group, the alkaline phosphatase activity was detected in the active Sertoli cells and Leydig cells (Figure 6H).

### Immunohistochemical analysis

**Figure 7:**
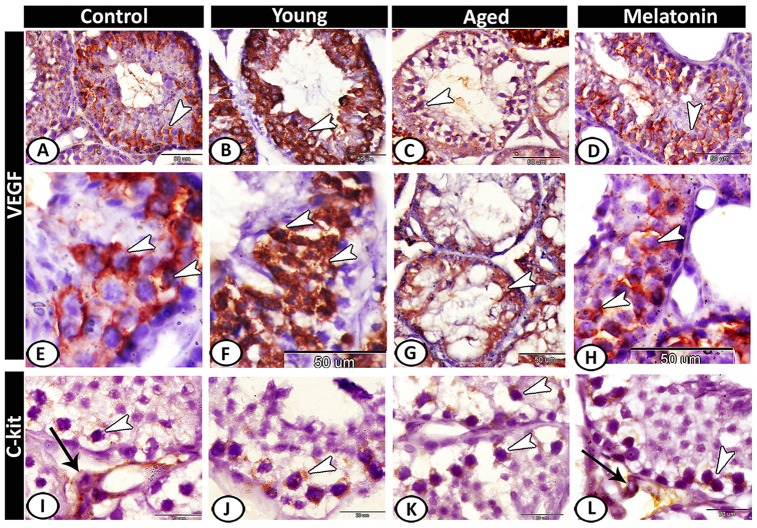
**Immunohistochemical expression of VEGF and c-kit in young and aged mice.** (**A**, **E**) VEGF expression in spermatocytes and spermatids (arrowheads) in the control group. (**B**, **F**) The young mutant mice showed overexpression of VEGF (arrowheads). (**C**, **G**) In aged mice, the reaction was weak and only the spermatocytes were immunostained (arrowheads). (**D**, **H**) In melatonin group, the reaction was concentrated in spermatocytes and spermatids (arrowheads). (**I**) c-kit expression was immunolocalized in spermatogonia (arrowhead) and interstitial cells (arrow) in the control group. (**J**, **K**) In young and aged mutant mice, the reactions were intense at the spermatogonial level (arrowheads). (**L**) The reaction in melatonin group was observed in spermatogonia (arrowhead) and Leydig cells (arrow).

VEGF expression in the control (Figure 7A, 7E) and melatonin group (Figure 7D, 7H) was concentrated in spermatocytes and spermatids. The young mutant mice showed overexpression of VEGF (Figure 7B, 7F). In aged mice, the reaction was weak and only the spermatocytes were immunostained (Figure 7C, 7G). CD117 or c-kit expression was immunolocalized in spermatogonia and interstitial cells in both the control (Figure 7I) and melatonin group (Figure 7L). In young (Figure 7J) and aged mutant mice (Figure 7K), the reaction was more intense at the spermatogonial level.

**Figure 8:**
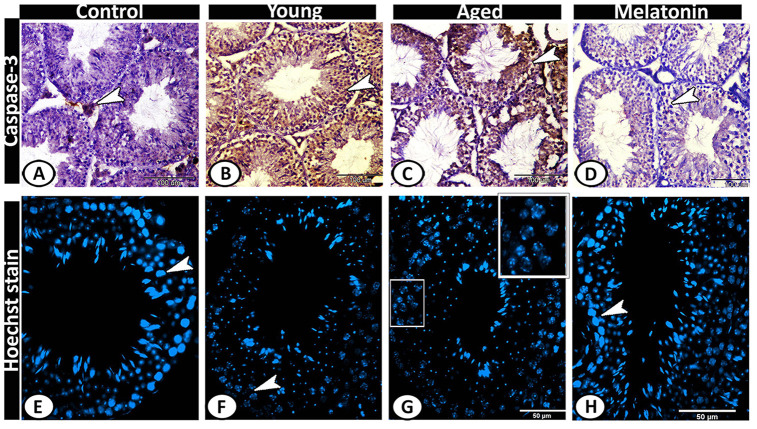
**Detection of testicular apoptosis of the young and aged mice.** (**A**) Caspase 3 was immunolocalized in Leydig cells (arrowhead) of the control group. (**B**, **C**) in both young and aged mutant mice showed overexpression of Caspase 3 in the seminiferous tubules and interstitium (arrowheads). (**D**) In the melatonin group, Caspase 3 immunostaining in seminiferous tubules showed a normal pattern of apoptosis (arrowhead). (**E**, **H**) The Hoechst immunofluorescent stain showed normal spermatogenic activity (arrowheads) in both the control and melatonin groups. (**F**, **G**) The spermatogenic cells of the young and aged mutant mice showed nuclear fragmentation, condensation, and hyperchromatasia (arrowhead, boxed areas).

Caspase 3 was immunolocalized in Leydig cells of the control group (Figure 8A), while its expression increased in both young and aged mutant mice to include all the seminiferous tubules and interstitium (Figure 8B, 8C). In the melatonin group, caspase 3 immunostaining in seminiferous tubules showed a normal pattern of apoptosis (Figure 8D). The Hoechst immunofluorescent stain showed normal spermatogenic activity in both the control and melatonin groups (Figure 8E, 8H), while the spermatogenic cells of the young and aged mutant mice showed apoptosis, nuclear fragmentation, condensation and hyperchromatasia especially in spermatogonia and spermatocytes (Figure 8F, 8G).

### Ultrastructural examination

**Figure 9:**
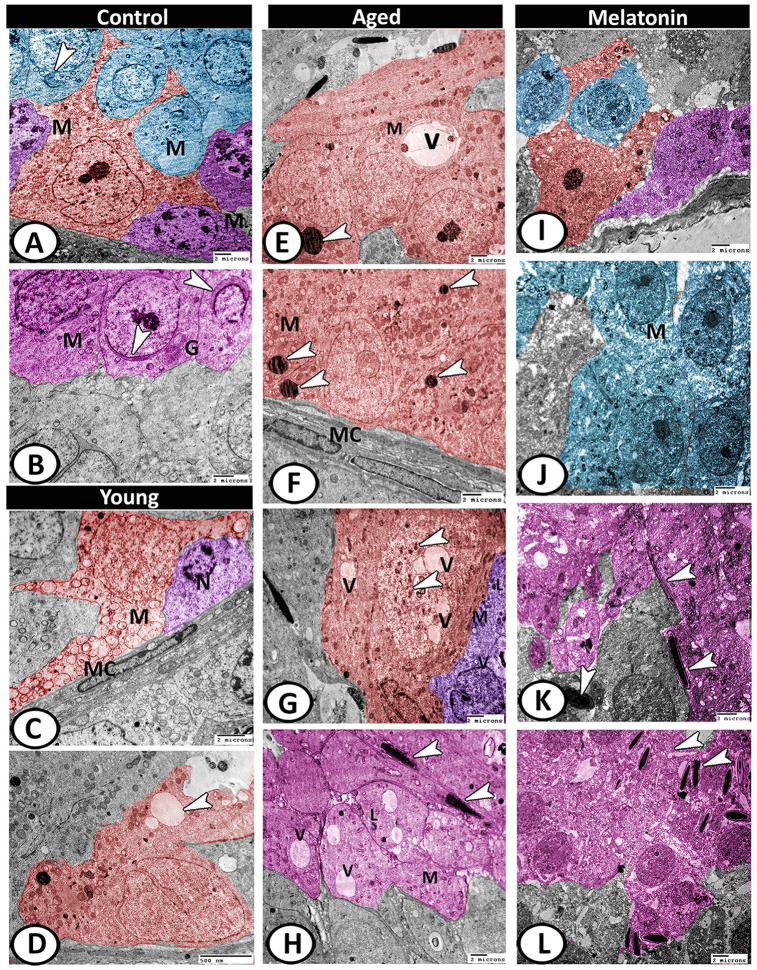
**Digitally colored TEM image showing the ultrastructural changes in the seminiferous tubules of young and aged mice.** (**A**) In the control group, the Sertoli cells (red) showed many cell processes and numerous mitochondria (M). Two spermatogonia populations (violet) were characterized by a network arrangement of nuclear chromatin and the cytoplasm contained mitochondria (M). The cytoplasm of primary spermatocytes (blue) was occupied by mitochondria (M)), and Golgi complex (arrowhead). (**B**) The spermatids (pink) characterized by the formation of an active acrosomal cap (arrowheads), presence of well-developed Golgi apparatus (G) and mitochondria (M). (**C**, **D**) In the young mutant mice, the Sertoli cells (red) showed degenerated mitochondria (M) and many vacuoles (arrowhead). The spermatogonia (violet) showed shrunken nuclei (N). Note, presence of myoid cells (MC) surrounding the seminiferous tubules. (**E**, **F**) In aged mice, the Sertoli cells' cytoplasm possessed vacuoles (V), mitochondrial metaplasia (M), lysosomes (arrowheads). Note, myoid cell (MC) showed no changes. (**G**) The cytoplasm of Sertoli cell (red) also contained phagocytosed materials (arrowheads) and vacuoles (V). The spermatogonia (violet) contained few mitochondria (M), vacuoles (V), and lysosomes (L). (**H**) The spermatids showed presence of many vacuoles (V), lysosomes (L), and the mitochondria (M) showed loss of their cristae. Many end pieces of degenerated sperms (arrowheads) could be demonstrated. (**I**) In melatonin group, the seminiferous tubules showed normal Sertoli cell (red), spermatogonia (violet) and spermatocytes (blue). (**J**) The spermatocytes (blue) contained numerous mitochondria (M) with some small vacuoles. (**K**, **L**) The differentiation of spermatids (pink) to normal sperms (arrowheads) was evident in melatonin group.

In the control group, the testes displayed normal architecture. The Sertoli cells were well-developed with many cell processes and oval indented large euchromatic nucleus and distinct nucleolus. Their cytoplasm was exhibited numerous mitochondria, sER profiles, and small lipid droplets. Two spermatogonia populations were observed that were characterized by a large nucleus with a network arrangement of chromatin. Their cytoplasm contained mitochondria and rER. The primary spermatocytes were characterized by their rounded configurations and distinct nuclear envelopes. Their cytoplasm was occupied by mitochondria, rER, and Golgi complex (Figure 9A). The spermatids characterized by active spermiogenesis in the form of the formation of active acrosomal cap, the presence of well-developed Golgi apparatus and mitochondria (Figure 9B). In the young mutant mice, the Sertoli cells showed degenerated mitochondria and many vacuoles. The spermatogonia showed shrunken and heterochromatic nuclei (Figure 9C, 9D).

With aging, the Sertoli cells exhibited euchromatic nucleus with irregular nuclear membranes and distinct nucleolus and their cytoplasm possessed vacuoles, mitochondrial metaplasia, and lysosomes (Figure 9E, 9F). The cytoplasm also contained lipid and phagocytosed materials. The spermatogonia contained few mitochondria, vacuoles, and lysosomes (Figure 9G). Myoid cell showed no changes (Figure 9F). The spermatids showed loss of acrosomal cap and the presence of many vacuoles of variable size, lysosomes, and the mitochondria showed loss of their cristae. Many end pieces of degenerated sperms could be demonstrated in the seminiferous tubules (Figure 9H). In the melatonin group, the seminiferous tubules preserve their normal architecture (Figure 9I). The spermatocytes contained numerous mitochondria with some small vacuoles (Figure 9J). The differentiation of spermatids to normal sperms was evident in the melatonin group (Figure 9K, 9L).

**Figure 10:**
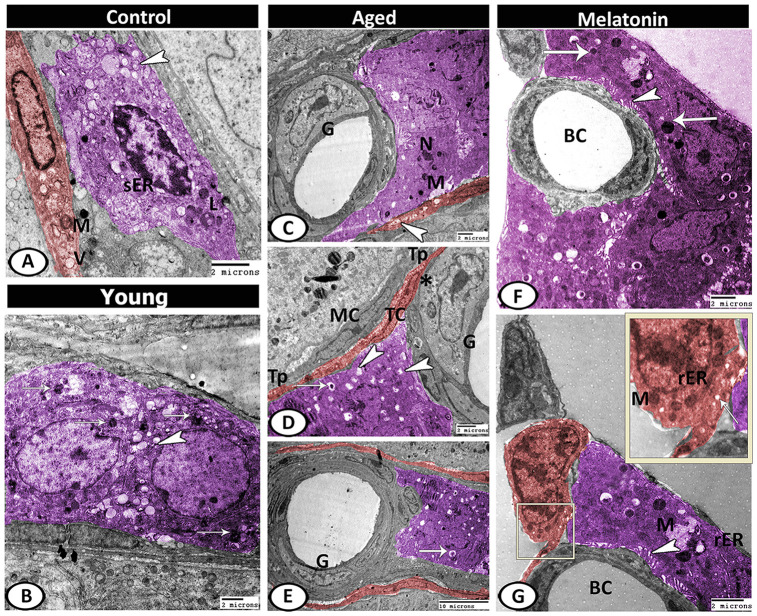
**Digitally colored TEM image showing the ultrastructural changes in the interstitial tissues of young and aged mice.** (**A**) In the control group, the Leydig cells (violet) contained lipid droplets (arrowhead), sER, lysosomes (L). Telocytes (TCs, red) contained secretory vesicles (V) and mitochondria (M). (**B**) In the young mutant mice, the Leydig cells (violet) characterized by an increase the number of lipid droplets (arrowhead) and lysosomes (arrows). (**C**) In aged mice, Leydig cell showed few mitochondria (M) and pyknotic nucleus (N). Note, the presence of TC (red) that contained many secretory vesicles (arrowhead) and the special type of blood vessel that characterized by the presence of large glomus cells (G). (**D**, **E**) Leydig cell (violet) contained many lipid droplets (arrowheads), and residual bodies (arrows). The glomus cell (G) was characterized by a high number of mitochondria (asterisk). The telocytes (red, TC) extended their telopodes (Tp) to establish close contact with Leydig cells, myoid cells (MC) and glomus cell (G). (**F**, **G**) In melatonin group, Leydig cells (violet) showed many mitochondria (M), secretory granules (arrows), rER, well-developed microvilli (arrowheads) that extended to the blood capillaries (BC). TCs (red) showed mitochondria (M), secretory vesicles (arrow) and rER.

Leydig cells in the control group contained lipid droplets, sER, lysosomes. Telocytes (TCs) were observed in the interstitium and characterized by the presence of oval euchromatic nucleus, secretory vesicles and mitochondria (Figure 10A). In the young mutant mice, the Leydig cells were characterized by increasing the number of lipid droplets and lysosomes (Figure 10B). With aging, Leydig cell cytoplasm appeared electron-dense with few mitochondria and sER, many lipid droplets, residual bodies and pyknotic nucleus. A special type of blood vessel was reported in the interstitial tissue that characterized by the presence of large glomus cells. The glomus cell was characterized by a large euchromatic nucleus with a thin rim of heterochromatin attached to the nuclear envelope and a central nucleolus. Its cytoplasm was electron-lucent and contained a high number of mitochondria and rER. The telocytes increased in number from 3.67 ± 0.57 in the young mice and 2.33 ± 0.5 in the mutant mice to 4.67 ± 0.58 in the aged ones (Supplementary Figure 1F), and distributed around the seminiferous tubules and blood vessels. These cells were characterized by their spindle-shaped cell bodies and the presence of two processes (telopodes). The telopodes contained many small secretory vesicles. Telocytes extended their telopodes to establish close contact with Leydig cells, myoid cells, and glomus cell (Figure 10C–10E).

In the melatonin group, Leydig cells showed an increase in the cellular and nuclear diameter compared to the aged mice. In addition, the cytoplasm showed many mitochondria, secretory granules, and many profiles of rER as well as an increase in the number and length of microvilli that extended to the blood vessels was the most remarkable feature. Telocytes showed high activity in the melatonin group in the form of the presence of well-developed mitochondria, secretory vesicles, and rER (Figure 10F, 10G).

## DISCUSSION

Retinoid-related nuclear orphan receptor (ROR) family is an essential transcription factors that regulate diverse biological processes including inflammation [32], lipid homeostasis [33], circadian rhythms [34], developmental and cellular metabolism [35], as well as innate immune response initiation against inflammation [20]. Expression of RORα nuclear receptors were reported in various tissues including testis, where a relationship between Sertoli cells functions and RORα was detected [36]. Furthermore, RORα expression and melatonin membrane receptors were recently detected in sheep [37] and goat [38] Leydig cells. In the present study, Retinoid-related orphan receptor (RORα)-deficient mice showed age-related progressive damage in the testis tubules and interstitium as well as a massive degeneration in spermatids that lead to decrease in the number of sperms. One possible mechanism to repair this damage is the administration of melatonin that induced the repair of many testicular defects and increased the number of mature sperms and ATPase activity.

It has been reported that melatonin receptors are identified on gonadotropins of the anterior pituitary gland [39] and can directly act at the gonadal level on testicular somatic cells [40], as it has a direct action on Leydig cells, acting as a local modulator of androgen secretion [41]. Moreover, in Sertoli cells, melatonin affects cellular growth and proliferation, energy metabolism, as well as oxidative state, and thus has an important role in regulation of spermatogenesis [31]. Melatonin also acts as a protective agent against many toxicants, irradiations, and diseases, and it is a powerful antioxidant acting as a free radical scavenger, being twice as efficient as vit E in eliminating the peroxyl radicals, inducing the anti-oxidant enzymes [42].

Previous studies on testis reported the protective effects of melatonin on reduction of non-enzymatic lipid oxidation of rat testicular mitochondria and microsomes [43], reduction of irradiation-induced damage of rat testes [44], treatment of oxidative testicular damage induced by 2-bromopropane through its ROS scavenger and antiapoptotic effect [45], minimizing the testicular ischemic injury of rats’ cellular organelles by preserving the DNA against oxidative damage [46], reducing of germ cell apoptosis and cadmium-induced cellular stress in testes [47], alleviation of testicular damage and attenuation of germ cell apoptosis in hyperlipidemic mice [48], removing the cigar smoke-induced testicular injury and restoring normal testicular tissue [49]. Melatonin dose of 10 mg/kg used in this study has also revealed significant anti-aging beneficial effects in SAM and wild-type mice [24, 26, 50]. Furthermore, therapeutic doses of the melatonin ranging from 5 mg to 50 mg/kg bw were previously reported [51]. Using the human equivalent dose formula, we calculated here that 10 mg/kg/day in mice corresponds to 1.62 mg/kg for adult human of about 60 kg bw, with about 97.3 mg/day. Moreover, many clinical trials have used melatonin doses of 50 mg to 300 mg/day and have reported substantial benefits with no side effects [52–54].

Our previous study [14] revealed a series of pathological changes in the testicular morphology in ROR-α-deficient mice that may reach to spermatogenesis arrest. As regards, the vacuolation in the spermatogenic epithelium in the aged mutant mice may be due to lipid peroxidation with consequent cell membrane damage. The present study demonstrated pyknotic nuclei in many spermatogonia of aged mice. Kroemer et al. [55] considered the nuclear pyknosis is a feature of apoptosis. However, Kumar et al. [56] reported that pyknosis is a pattern of nuclear changes related to cell necrosis and is characterized by nuclear shrinkage with increased basophilia, where condensation of DNA to a solid shrunken mass is occurred.

Caspase 3 showed massive apoptosis in aged mutant mice, producing an evident germ cell loss. These testicular changes were attributed to the increase in tissue oxidative stress and to the fall in androgen levels [57]. Melatonin supplementation however, showed a normal pattern of apoptosis that was confirmed by Hoechst immunofluorescent stain, assuring the anti-apoptotic effect of melatonin in testis [58]. In many mammals, the aging of males results in a reduction in the number of spermatogenic cells and consequently decrease in the daily sperm production. Our results revealed a mosaic appearance of the seminiferous tubules in the aged mutant mice; the atrophic tubules are often observed adjacent to tubules displaying normal spermatogenesis. These results are in line with that observed in aged humans and rodents [59–61]. In aged rats, the atrophy is due to a decrease in Sertoli cells numbers and also number of compacted spermatids per Sertoli cell [62].

Sertoli cells are suggested to be among the most susceptible cells to age-induced dysfunctions in the male reproductive system [63]. These cells are termed as testicular “nurse cells”. This define arise because the interactions between these cells and germ cells are essential for normal spermatogenesis, and structural and/or functional changes of Sertoli cells with aging could affect highly on spermatogenesis [64]. In mutant mice, Sertoli cells showed vacuolations that increased in old age. Sertoli cells vacuolation is the key feature of testicular damage that may result in sloughing and death of germ cells [65]. Also, the degenerated germ cells that phagocytosed by Sertoli cells result in lipid droplets accumulation in the cytoplasm of Sertoli cells. The decrease in the number of Sertoli cells in aging usually proportionate with the decrease in germ cell numbers. Furthermore, other study suggested the link between the reductions of Sertoli cells number and decrease of seminiferous epithelial volume [60].

The TEM revealed that the cytoplasm of spermatids contain numerous mitochondria that correlated with the high activity of ATPase in the control group. Increase in ATPase and alkaline phosphatase activity in mutant mice may be due to degeneration of germ cells and/ or phagocytosis of degenerating germ cells by Sertoli cells in agreement with Delhumeau-Ongray et al. [66].

One of the causes of the testicular involution during aging is vascular alteration or defective microvascularization in the peritubular capillaries [60]. Vascular endothelial growth factor (VEGF) in aged mutant mice showed low expression in seminiferous tubules. Sadoun and Reed [67] reported an age-related progressive impairment in mechanisms of the vascular repair, associated with a decrease in the circulating androgen levels in men, which is the main regulator of the angiogenesis. Aged mammals exhibited remarkable impairment in angiogenesis after vascular and ischemic injuries. These impairments are due to a reduction in the proangiogenic growth factors and cytokines production in aged animals, specifically VEGF.

It is well known that c-kit is a type III receptor tyrosine kinase, which participates in many intracellular signaling whereby it is essentially considered a stem cell factor receptor. The present study revealed that c-kit expression was more intense at the spermatogonial level in the aged mutant mice. Gude, et al. [68] reported that c-kit expression increases in response to cellular stress. On the other hand, Marino, et al. [69] suggested that the intense expression of c-kit in old age might indicate the active cellular regeneration to counteract the aging effects on the organ structure and function.

Our results showed alteration in testis interstitium of aged mutant mice in the form of Leydig cells hyperplasia, increase in the collagen fibers, presence of modified blood vessels (glomus), many lipid droplets, and residual bodies. The abundance occurrence of collagen fibers in the interstitial tissues with modifications in the blood vessels could contribute to the decline in testosterone secretion by Leydig cells with advancing in age. A significant increase in Leydig cells number were evident in the aged mice, similar to that reported by Ichihara et al. [70], where the number of Leydig cells doubled with age in rats, however; their average volume decreased. The observed hyperplasia of Leydig cell could be a compensatory mechanism to maintain the normal androgen level. The accumulation of lipid droplets was previously reported in Leydig cells of old mice [71], horse [72], cat [73] and rat [70]. Our study revealed the presence of mast cells in interstitium of aged mice that may be related to infertility based on the study of Meineke et al. [74] who recorded higher populations of mast cells in infertile men than in healthy ones.

Previous studies reported the age-related reduction of germinal cells number in seminiferous tubule in many mammals [75, 76], which results in reduction of seminiferous tubules diameter [77] and epithelium vacuolization [78], similar to findings observed in this study. Interestingly, melatonin group showed normal spermatogenesis and preserved normal germ cell counts as well as nuclear and cytoplasmic features of the testicular cells. These results may be attributed to that melatonin is expressed in the testicular cells [79] and induce the spermatogonial differentiation to functional haploid germ cells [37]. Melatonin also increases the activity of plasminogen in ram sperms [80].

Melatonin increases GATA-4 expression to induce testosterone synthesis by Leydig cells, promoting consequently more active meiosis and spermatid differentiation [38]. In this regard, the present study revealed an increase in the cellular activity of Leydig cells in the melatonin group, associated with an increase in the number of mitochondria and secretory granules. Moreover, melatonin receptors (MT1 and MT2) were identified in bovine and rat Sertoli cells [81, 82], where melatonin regulates Sertoli cells metabolism, proliferation and consequently affect spermatogenesis. Furthermore, melatonin boosts proliferation of spermatogonial stem cells through inducing production of glial cell line-derived neurotrophic factor in Sertoli cells [83].

Telocytes were observed in the mouse's testis [14, 84]. The authors added that TCs are an important component of testicular interstitium in the mouse, possibly involved in maintaining its microenvironment as well as having contractile and secretory functions. Moreover, Hasirci et al. [85] confirmed that the distribution and number of TCs may affect spermatogenesis. The present study showed that TCs increase in number in the aged mutant mice and distributed around seminiferous tubules and blood vessels. These results match with the study of Popescu et al. [86] in aging human heart who found that TCs form an extensive 3D network and occupying a large volume in the interstitium. Hussein and Mokhtar [87] concluded that TCs have a role in the angiogenesis. So, their abundant presence in aged mice may be a compensatory effort to repair the vascular damage. Therefore, these cells may be an essential target cell type for treatment of testicular interstitium disorders.

In conclusion, lack of ROR-α transcription factor results in progressive diverse testicular damage confirming by that the crucial role of this protein in regulation of testicular structure and functions. This damage increases with the advancement of age; however; melatonin supplementation improves testicular architecture and properties in aged mice. Thus, melatonin use should be highly recommended as a medical therapy for various testicular dysfunctions.

## MATERIALS AND METHODS

### Animals

Both wild-type (WT) C57BL/6 and heterozygous B6.C3 (Cg)-RORa^sg/J (RORa+/sg)^ mice were obtained from Jackson Laboratory (Bar Harbor, ME, USA). Crossing of heterozygotes was performed to obtain homozygous stagger mice (RORa^sg/sg^). Animals were kept in a specific pathogen-free barrier zone at Granada University facility at summer season under controlled temperature (22 ± 1°C) and 12:12-hour light-dark cycle (lights on at 08:00 hour), and fed with rodent chow containing melatonin or placebo, in addition to unlimited access to water.

The animals were categorized into 4 groups (*n* = 10 mice per group); control (WT mice of 10-12 weeks-old), young mutant (mature young ROR^-/-^ mice of 10-12 weeks-old), aged (aged ROR^-/-^ mice of 9-10 months-old), and aged treated with melatonin (aged ROR^-/-^ mice of 9-10 months-old fed on the same rodent chow supplemented with melatonin in a concentration that allow a daily intake of 10 mg/kg b.w./mouse for 4 months before sacrificing). Studying of testicular aging was investigated here in the mutant mice at the age of 9-10 months in accordance with previous study, which reported that about half of the RORα-deficient mice die shortly after weaning and the remaining could reach adulthood [13]. Wild-type mice were used in this study at the age of 3 months to compare findings with those of mutant mice at the same age for clarifying the role of RORα on testicular structure and function. Moreover, we couldn't use WT-mice at age of 9 months for aging study as this strain of mice could survive for about 30 months and thus 9 months of age is not considered aged.

All experiments were performed according to Granada’s University Ethical Committee, Spanish law for animal experimentation (R.D. 53/2013) and European Convention for the Protection of Vertebrate Animals Used for Experimental and Other Scientific Purposes (CETS #123).

### Histological and histochemical analyses

Animals were weighted and then were transcardially perfused with saline and then with a freshly prepared trump's fixative solution of 3.7% formaldehyde and 1% glutaraldehyde in saline buffer. This step was performed under complete anesthesia through intraperitoneal injection of equithensin (1 ml/kg). Following that, the testes were dissected from excessive tissue and weighted, and the ratio of testicular weight to body weight was analyzed.

For histological examination, small samples (1x1x0.5 cm) were collected from testis of experimental groups and were immediately fixed in Bouin's solution for 22 hours. The fixed samples were dehydrated in ethanol, cleared in methyl benzoate and then embedded in paraffin wax. Transverse sections of 5-8 μm thickness were obtained by a Richert Leica Microtome (RM 2125, Germany), stained with Harris hematoxylin and eosin [88] and Crossmon’s trichrome [89].

For demonstrating carbohydrate and protein content, sections were stained by histochemical Periodic Acid-Schiff (PAS) [90] and bromophenol blue [91] stains respectively. Moreover, some sections were stained for lipid content by sudan black B [92].

For enzyme histochemistry, activity of ATPase was detected at pH 4.2 [93], while activity of alkaline phosphatase was investigated by the Gomori calcium method [94]. The sections were examined using a Letiz Dialux 20 Microscope, and images were acquired by a Canon digital camera (Candison Powershot A95).

### Immunofluorescent analysis of nuclear apoptosis

For detection of apoptotic nuclei, paraffin sections of 4 μm thick were immersed in xylene for dewaxation, and in a descending series of ethanol for rehydration, and then were washed with distilled water. The sections were left for air-drying following with rinsing in PBS 1X (2 × 5 min) and finally were stained with 33258 Hoechst dye (H6024, Sigma-Aldrich, Spain) for 1 hr. The sections after staining were washed in PBS 1X (5 × 5 min), air-dried, mounted, and examined with LEICA DM5500B fluorescent microscope [95].

### Semithin sections and transmission electron microscopy (TEM) preparations

Small samples of the testis of experimental groups were immersed in a mixture of 3% paraformaldehyde–glutaraldehyde fixative and left overnight for preservation [96]. The samples were then washed in 0.1 M phosphate buffer and immersed in 1% osmium tetroxide in 0.1 M sodium cacodylate buffer at pH 7.3. Following that, samples were passed in a graded series of ethanol followed by propylene oxide for dehydration, and finally embedded in Araldite.

Semithin sections (1 μm thickness) were cut by Richert Ultracuts (Leica, Germany) and were stained with toluidine blue for light microscopy. While, ultrathin sections (70 nm) were cut using Ultrotom VRV (LKB Bromma, Germany). The sections were stained with uranyl acetate and lead citrate [97], and were examined at the Electron Microscopy Unit of Assiut University, Egypt by JEOL 100CX II transmission electron microscope.

### Digital colored images

Digital colored images were designed in order to increase the visual contrast between several structures (Sertoli cells, Spermatogonia, Leydig cells, telocytes, etc.) within the same electron micrograph to be easily visible to the reader. All of these structures were carefully hand colored using Adobe Photoshop software version 6.

### Immunohistochemical analysis

Representative paraffin sections of experimental groups were rehydrated in graded ethanol after deparaffinization with xylene, followed by washing twice with distilled water. The sections were heated in 0.01M sodium citrate buffer (pH 6.0) in a microwave oven for 15 min to increase epitope exposure. Sections were washed with PBS after cooling, and then blocked for 1 h with 10% bovine serum albumin (BSA) in TBST buffer (0.05% Tween 20, 20 mM Tris-buffered saline, pH 7.5) at room temperature. After blocking, the sections were incubated at 4ºC overnight with diluted (1:400) polyclonal antibodies against caspase-3, vascular endothelial growth factor (VEGF), and CD117 (C-kit, stem cell growth factor receptor). Visualization of the antibodies was performed using SABC Kit Elite as well as 0.05% 3, 3-diaminobenzidine tetrachloride (Sigma) in PBS, including 0.01% H_2_O_2_ for 2 min. The representative sections were then counterstained with hematoxylin and mounted. Specificity of the antibodies was examined through replacement of the primary antibodies by 1% BSA.

### Morphometrical and statistical analyses

Light microscopical and TEM images were used for morphometrical analyses of the testes of the experimental groups with the help of Leica Q 500MC image analyzer (Leica, Germany). Different morphometrical measurements were performed on ten randomly selected sections for each animal. These measurements include:

- Number of seminiferous tubules/ 400 μm^2^ using 20X objective.
- The diameter of seminiferous tubules (μm) using 20X objective.
- Height of the epithelium of seminiferous tubules (μm) using 40X objective.
- The volume percentage of interstitial tissues (%) using 20X objective.
- Number of Leydig cell/ 50 μm^2^ using 100X objective.
- Number of telocytes/ 50 μm^2^ using 100X objective.

The statistical analysis was done using Graph Pad Prism software (GraphPad, San Diego, CA, USA), and the data are presented as the means ± *SEM*. One-way ANOVA with a *Tukey’s* post hoc test was used for statistical comparison between experimental groups. Differences were considered significant when *P* < 0.05.

## Supplementary Material

Supplementary Figure 1
