# Supplementary material for: Lack of retinoid acid receptor-related orphan receptor alpha accelerates and melatonin supplementation prevents testicular aging
Source: Aging (Albany NY). 2020 Jul 9;12(13):12648–68. doi: 10.18632/aging.103654 (PMC7377884; doi:10.18632/aging.103654)
Supplement: Supplementary Figure 1 [file aging-12-103654-s001..pdf]

SUPPLEMENTARY FIGURE

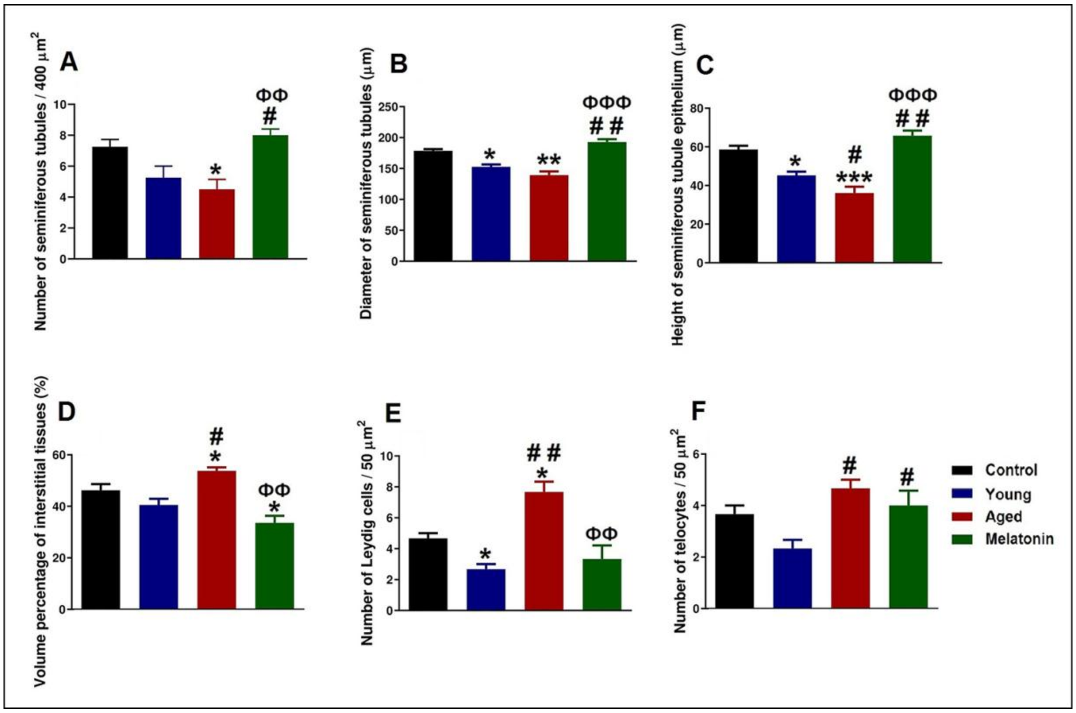

Supplementary Figure 1. Morphometrical analysis of seminiferous tubules number (**A**) and diameter (**B**), height of seminiferous tubules epithelium (**C**), volume percentage of the interstitial tissues (**D**), number of Leydig cells (**E**) and teloctyes (**F**) in young and aged mice. * *p*<.05, ** *p*<.01 and *** *p*<.001 *vs.* Control; # *p*<.05 and ## *p*<.01 *vs.* Young; ΦΦ *p*<.01 and ΦΦΦ *p*<.001 *vs.* Aged.
